# Oxygenation-sensitive cardiovascular magnetic resonance

**DOI:** 10.1186/1532-429X-15-43

**Published:** 2013-05-24

**Authors:** Matthias G Friedrich, Theodoros D Karamitsos

**Affiliations:** 1 Montreal Heart Institute, Departments of Cardiology and Radiology, Université de Montréal, Montreal, QC, Canada; 2 Departments of Cardiac Sciences and Radiology, University of Calgary, Calgary, Canada; 3 Oxford Centre for Clinical Magnetic Resonance Research, Division of Cardiovascular Medicine, Radcliffe Department of Medicine, University of Oxford, John Radcliffe Hospital, Oxford, UK

**Keywords:** Cardiovascular magnetic resonance, Blood-oxygen level-dependent, Microcirculation, Ischemia, Oxygenation

## Abstract

Oxygenation-sensitive cardiovascular magnetic resonance (CMR) is a non-contrast technique that allows the non-invasive assessment of myocardial oxygenation. It capitalizes on the fact that deoxygenated hemoglobin in blood can act as an intrinsic contrast agent, changing proton signals in a fashion that can be imaged to reflect the level of blood oxygenation. Increases in O_2_ saturation increase the BOLD imaging signal (T2 or T2*), whereas decreases diminish it. This review presents the basic concepts and limitations of the BOLD technique, and summarizes the preclinical and clinical studies in the assessment of myocardial oxygenation with a focus on recent advances. Finally, it provides future directions and a brief look at emerging techniques of this evolving CMR field.

## Review

### Contrast Generation in Oxygenation-Sensitive CMR

It was Linus Pauling who first described an effect of the oxygenation state of hemoglobin on its magnetic properties [1]. With respect to its behavior during magnetic resonance experiments, the de-oxygenation of hemoglobin causes the molecule to act as an intrinsic paramagnetic contrast agent resulting in pronounced spin-spin interaction. This accelerates the decay of transverse magnetization and thus shortens the spin-spin relaxation time. The term T2* describes the rate of loss of the transverse signal when using sequences without refocusing radiofrequency pulses. Regional iron deposition, hemoglobin degradation products in tissue hemorrhage or deoxygenated hemoglobin accelerate this process by their paramagnetic properties, a phenomenon called the BOLD (Blood-Oxygen-Level-Dependent) effect. This phenomenon can be exploited to perform “BOLD-sensitive” or, depending on the context, “oxygenation-sensitive” imaging with the aim to detect changes in tissue oxygenation.

In 1990, Ogawa et al. demonstrated that oxygenation-sensitive MR can be used to detect consequences of very small blood flow changes in the brain resulting from external stimuli [2]. In humans, typically several averages and color-coded maps are used to visualize these changes [3], a technique which is widely known as functional brain MR. Accordingly, myocardial deoxygenation or ischemia is characterized by a net relative increase of de-oxygenated hemoglobin in the capillary blood and thus leads to T2* shortening, which can be visualized by T2* maps or by a regional signal loss in “T2*-weighted” MR images (i.e. images acquired by protocols sensitive to decreased regional field homogeneity). Conversely, a decrease of the proportion of de-oxygenated hemoglobin (for example by inducing vasodilation without a matching increase of myocardial oxygen demand) causes a relative decrease of de-oxygenated hemoglobin and leads to an increase of T2* in this territory and hence to an increased signal intensity in oxygenation-sensitive images.

It is important to keep in mind that the observed changes reflect changes in (mainly the venous compartment of the) capillary bed and therefore strictly do not represent the actual cell (i.e. cardiomyocytes). Yet the oxygenation of the capillary blood directly reflects the balance of oxygen supply and demand and therefore can be understood as a direct marker of tissue oxygenation.

### Technical aspects of oxygenation-sensitive CMR

While image quality is frequently affected in echo-planar sequences [4] and other, T2-weighted sequences [5] by motion or susceptibility artifacts (especially along the lung-heart interface), steady-state-free-precession sequences have shown promising results, allowing for a simultaneous acquisition of morphological, functional and oxygenation-sensitive data of the heart [6]. Of note, the oxygenation-sensitive contrast in images acquired by this sequence is directly dependent on the repetition time TR [7]. With an appropriate TR, the obtained image quality is much more consistent when compared to other BOLD-sensitive sequences, though at the expense of reduced sensitivity to the BOLD effect and thus lower “oxygenation contrast”. Further studies may have to fine-tune the balance between sensitivity to oxygenation changes and susceptibility artifacts.

Importantly, the magnitude of the BOLD effect (i.e. the measurable effect size) largely depends on the strength of the static magnetic field. While the overall susceptibility to field inhomogeneity at higher field strengths may cause artifacts, it also accounts for a higher sensitivity to changes induced by paramagnetic effects. Higher field strengths thus improve the sensitivity of MR to the BOLD effect. Data by Dharmakumar et al. indicate that the sensitivity to detect changes in myocardial oxygenation may increase by a factor of about 2.5 when moving from 1.5T to 3T [8].

With respect to a clinical application in patients with suspected myocardial ischemia, it is very important to be aware of the significant limitations of current diagnostic techniques to verify the hemodynamic relevance of coronary artery disease. Because of a lack of diagnostic targets on the (cellular) level of actual ischemia, imaging techniques use surrogate markers such as stress-inducible dysfunction (echocardiography), changes of blood inflow characteristics (echocardiography, first-pass perfusion CMR) or metabolic changes (nuclear cardiology techniques). All these are surrogates and thus cannot directly reflect an ischemic response of myocardial tissue, while oxygenation-sensitive CMR offers exactly that.

### Experimental and pre-clinical studies

In 1993, Atalay et al. used a Langendorff heart model to show that the signal intensity in images sensitive to the BOLD effect (in that paper called “susceptibility-dependent”) is closely correlated to blood oxygenation [9]. Wendland et al. induced hypoxia in rats and demonstrated changes in the blood and the myocardium caused by the BOLD effect [10].

In July 1996, Niemi et al. observed in healthy volunteers, that the increase of myocardial blood flow as induced by the infusion of the vasodilator dipyridamole was closely correlated with a myocardial signal intensity increase in images obtained by a T2*-sensitive single-shot echo planar imaging (EPI) sequence [11]. Yet, another study, published in the same journal issue by Li et al. shed further light on the relationship of blood flow and oxygenation and thus the precise determinants of the observed signal intensity changes. Using an oxygenation-sensitive gradient echo sequence, they could demonstrate that signal intensity changes primarily reflect changes of myocardial oxygenation and not changes of blood flow [12]: While a simultaneous, “balanced” increase of flow and demand (using dobutamine) did not significantly alter the signal, a non-balanced blood flow increase without accompanying increase of oxygen consumption (dipyridamole) lead to an increase of the myocardial signal intensity (hence oxygenation). This experiment helped to exclude relevant confounding effects of blood volume and flow on the ability of BOLD-sensitive CMR to assess changes of blood (capillary) oxygenation.

**Figure 1:**
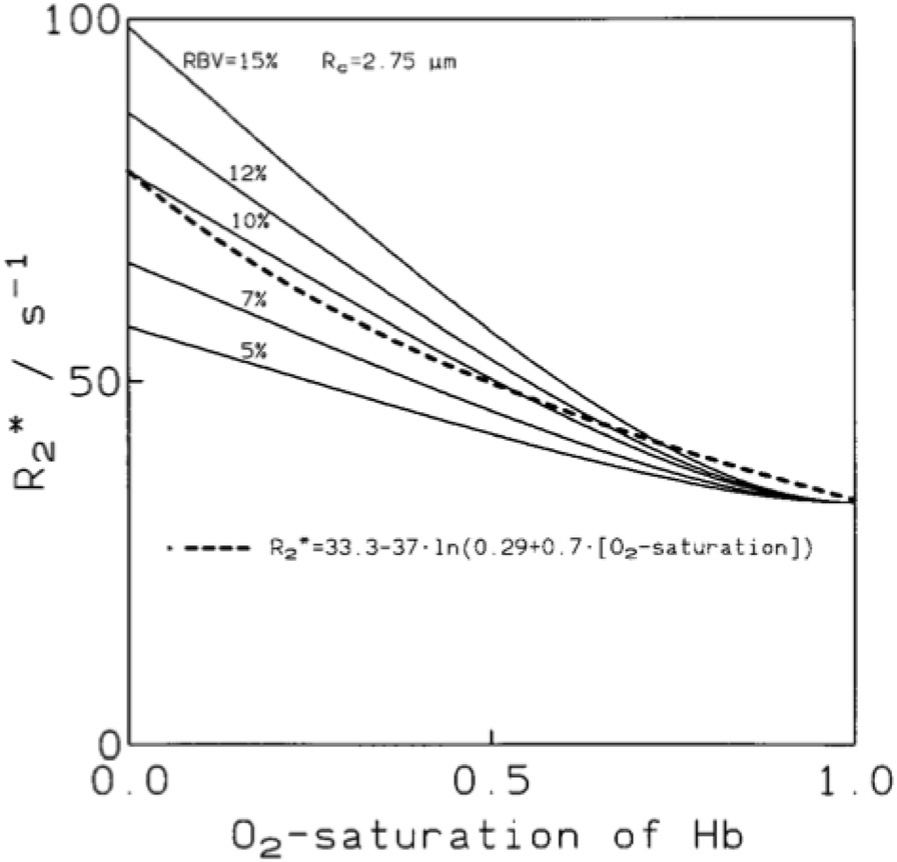
Relationship between oxygenation and T2* (from [[18]**]).**

After extensive theoretical considerations on oxygenation-sensitive CMR [13] (Figure 1), Bauer et al. applied the approach of T2* mapping to visualize changes of myocardial oxygenation following dipyridamole in volunteers [14]. As an important step, signal intensity changes in the myocardium of dogs induced by vasodilation could be validated against microspheres [15]. With respect to its potential use to assess the relevance of coronary artery stenosis, an SSFP technique was used to detect a lack of signal intensity changes in the perfusion bed of experimentally (partially) occluded coronary arteries and demonstrated the ability of oxygenation-sensitive CMR to detect consequences of significantly reduced blood flow for myocardial oxygenation [16]. Tsaftaris et al found that, in severe coronary artery stenosis, the ratio of the systolic over the diastolic signal intensity in oxygenation-sensitive cine CMR images is already altered at rest, i.e. without any provocative testing [17].

**Figure 2:**
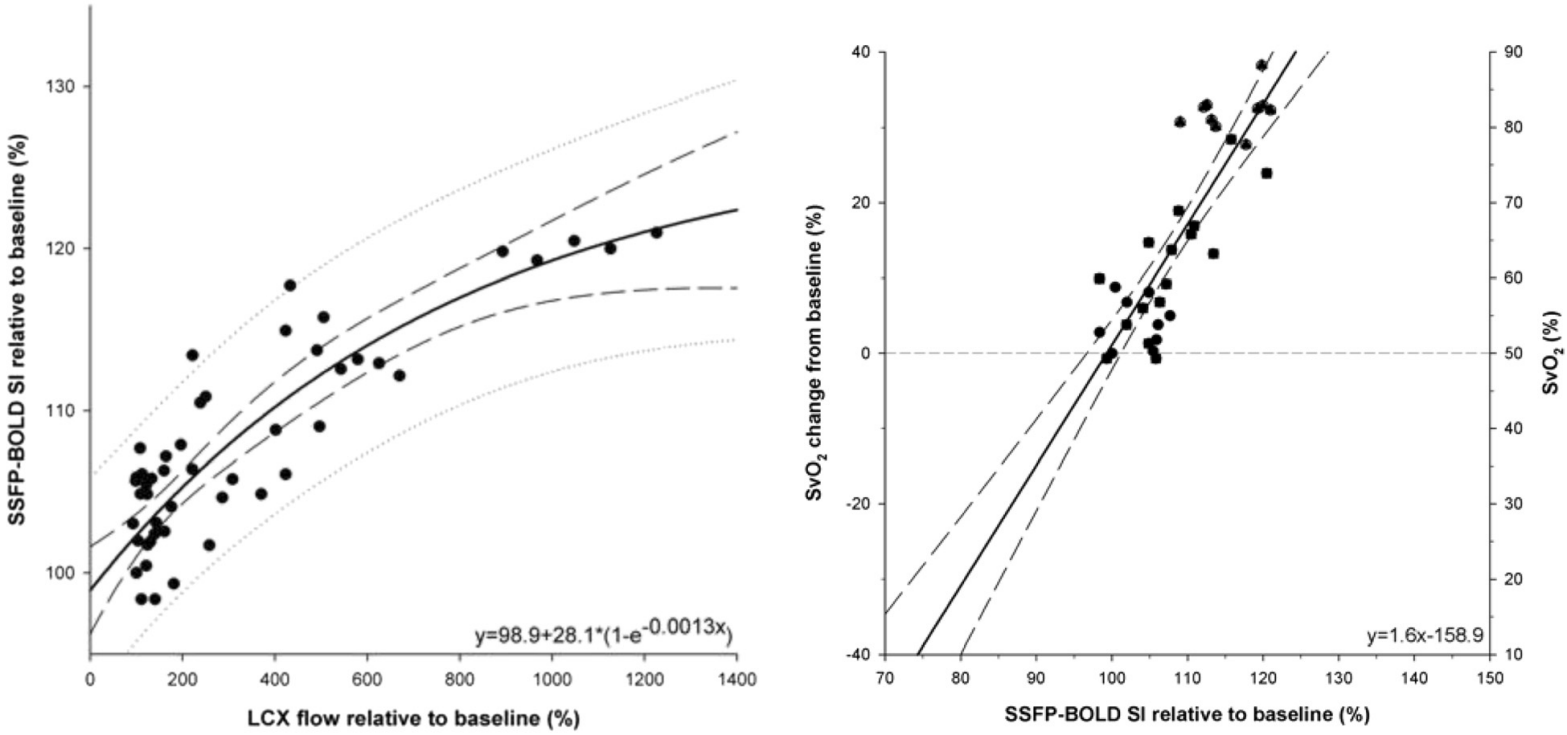
**Correlation of changes of myocardial oxygenation during Adenosine infusion with blood flow and coronary sinus oxygenation.** Note the exponential relationship between flow and signal intensity as opposed to the linear relationship with changes of coronary sinus blood oxygenation, showing that signal intensity changes reflect changes of oxygen and not blood flow (modified from [19].

While previous studies did not always distinguish between the impact of blood flow, perfusion, blood volume on the observed signal intensity in oxygenation-sensitive images, Voehringer et al. could show in an animal model with intracoronary injection of vasoactive substances that the signal intensities observed in oxygenation-sensitive images of the myocardium indeed reflect myocardial blood oxygenation and not blood flow [19] (Figure 2), validating previous data by Li et al. [12] by blood oxygen measurements.

Oxygenation-sensitive CMR was also applied to assess endothelial function in the skeletal muscle [20], but similar studies have not been performed yet in the heart.

Expanding the scope of potential applications, Zheng et al. observed a close inverse correlation between changes of oxygenation and changes of oxygen extraction in a dog model with dipyridamole-induced hyperemia and proposed measuring the actual oxygen extraction fraction by oxygenation-sensitive CMR [21]. The change of the myocardial consumption rate has been measured in volunteers using a single breath-hold T2 mapping technique [22].

Very recent studies provide preliminary evidence that the clinical utility of oxygenation-sensitive CMR could be expanded to include vascular function. Günsch et al. used oxygenation-sensitive CMR to demonstrate the impact of breathing maneuvers on myocardial oxygenation [23]. This approach may offer a novel diagnostic approach to assess both, coronary vascular function and the hemodynamic relevance of coronary artery stenoses.

### Clinical studies

#### Brain

**Figure 3:**
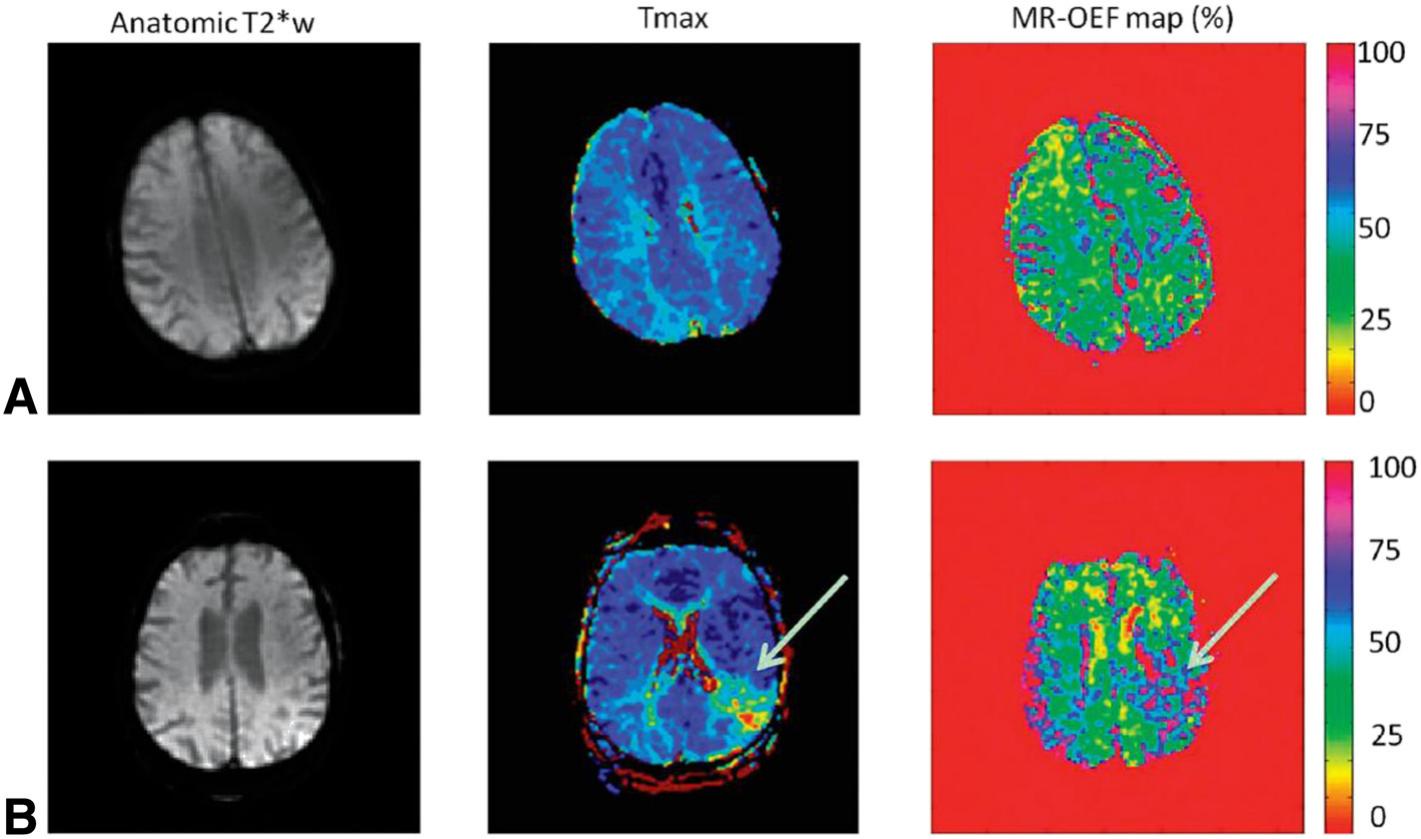
**Parametric maps obtained with a quantitative BOLD approach by using a multiecho gradient-echo and spin-echo echo-planar imaging method in a healthy subject (A) and a patient with stroke (B).** High oxygen-extraction fraction (OEF) can be observed in the affected region of the patient with stroke (white arrow). This method holds promise to evaluate the brain oxygenation status in a rapid fashion, which is critical in the acute stroke work-up (from [27]).

BOLD imaging has been used for many years in the field of cognitive neuroscience. Both simple T2* estimates of oxygenation and more advanced quantitative BOLD techniques have been used to detect oxygenation changes in different brain disorders (Figure 3). T2* measurements have been proposed to delineate the penumbra in acute stroke [24,25]. Furthermore, BOLD imaging has the potential to assess oxygenation status of brain tumors [26]. Many other brain disorders such as Alzheimer disease, Parkinson disease and Huntington disease have been associated with alterations in cerebral oxygen metabolism [27]. A detailed description of these studies is beyond the scope of this article.

#### Cardiac studies – detection of CAD

**Figure 4:**
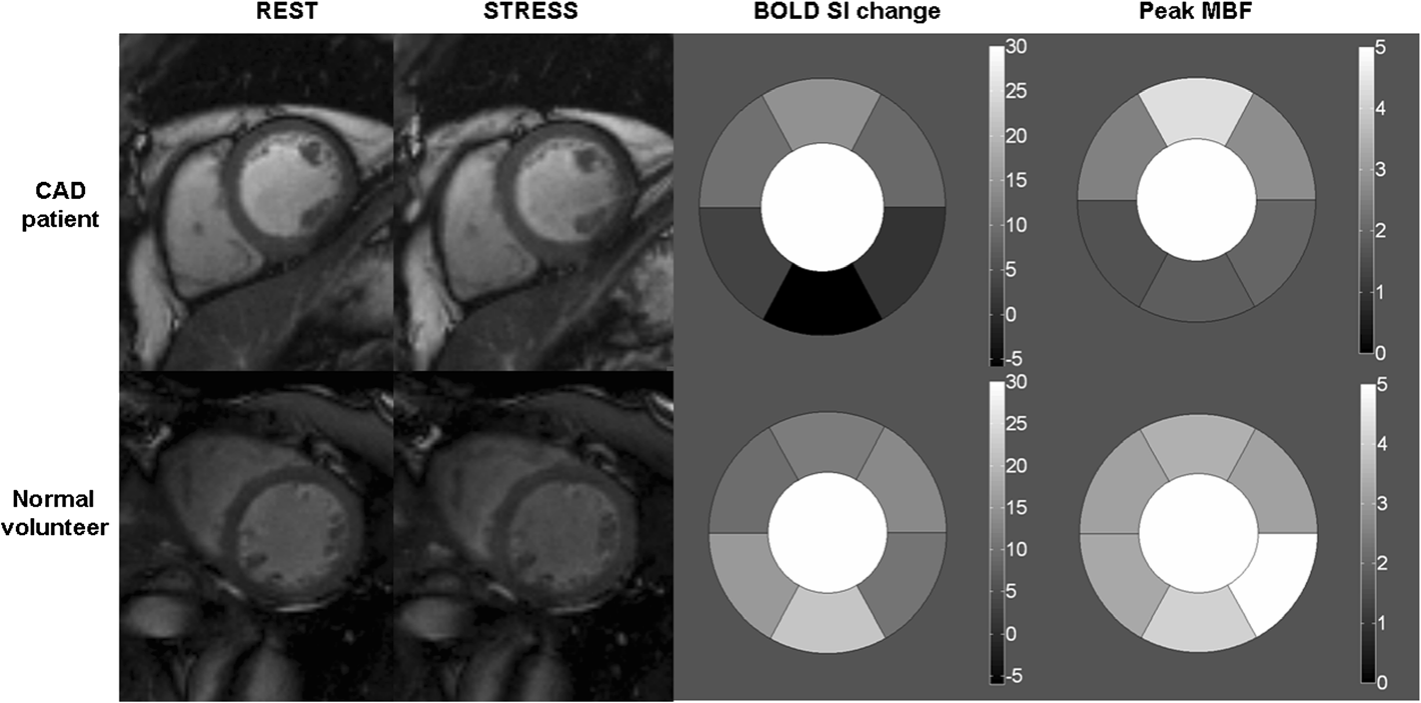
**Oxygenation-sensitive imaging in coronary artery disease: Note the regional abnormality reflecting a lack of signal intensity change in a coronary territory subtended by a stenotic artery (from [**[32]**]).**

**Figure 5:**
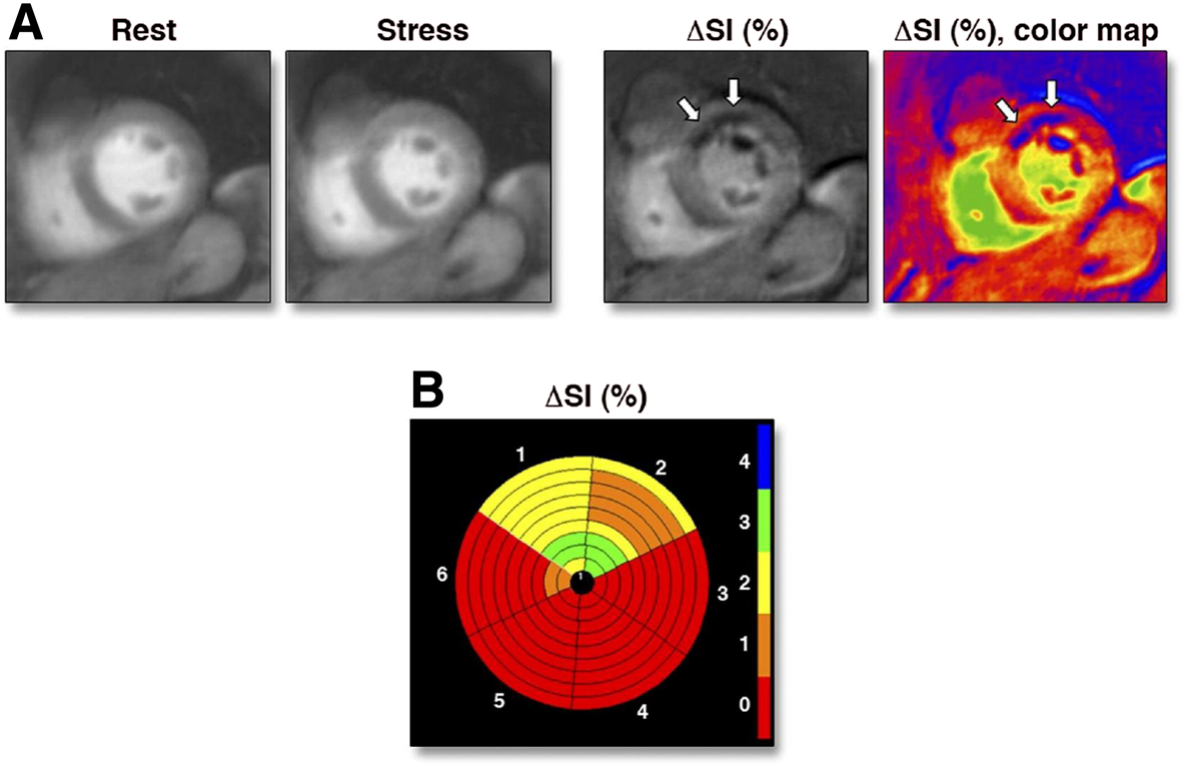
**Rest and Stress BOLD Images With Corresponding Subtraction Images. (A)** The BOLD CMR images at rest and during adenosine stress together with the corresponding subtraction image and its parametric color map. A stress-induced signal loss in the anterior myocardial segment can be seen (white arrows). **(B)** Bull’s eye plot of the complete 3-dimensional data set of subtracted BOLD images showing a stress-inducible signal loss in the anterior segments. The color scale indicates the transmurality of the stress-induced signal loss (0 = no signal loss, 1 = 1% to 25%, 2 = 26% to 50%, 3 = 51% to 75%, 4 = 76% to 100% transmurality). BOLD = blood oxygen level–dependent; CMR = cardiovascular magnetic resonance; ΔSI = relative signal intensity changes of blood oxygen level–dependent cardiovascular magnetic resonance (from [36]).

After the successful demonstration of the BOLD effect in preclinical animal studies, the next step was the evaluation of oxygenation-sensitive CMR in normal volunteers and patients with known or suspected coronary artery disease (CAD). After a small pilot study [28], Wacker and colleagues used a segmented gradient echo pulse sequence for T2* myocardial measurements at rest and during dipyridamole stress in 16 normal controls and 16 patients with single vessel CAD [29]. During dipyridamole infusion, T2* increased significantly from 35 ± 3ms to 40 ± 4ms in healthy volunteers (i.e., by 10 ± 5%; p = 0.01). In contrast, myocardial segments supplied by a stenotic coronary artery showed a reduced T2* at rest and a modest response to vasodilation. Two patients with proximal stenosis of left anterior descending coronary artery were re-investigated after revascularization. Following intervention, the T2* homogeneity of the myocardium was improved. In another 1.5T study, Friedrich and colleagues compared T2* oxygenation sensitive CMR with single-photon emission computed tomography (SPECT) [30]. This study included 25 patients with exercise-induced angina and assessed oxygenation SI changes at rest and during adenosine stress in a single mid-ventricular short axis. Myocardial segments subtended by vessels with >75% stenosis showed a significant decrease in signal intensity compared to segments with no stenosis. Receiver operator characteristics analysis of both oxygenation-sensitive CMR and thallium SPECT as related to quantitative coronary angiography revealed similar areas under the curve, 0.66 and 0.73, respectively. The investigators concluded that oxygenation-sensitive CMR is a promising technique to detect significant CAD. They also outlined the work that has to be done to improve the technique such as the need to increase spatial coverage and signal changes but also reduce artifacts. More recently, Manka and colleagues performed T2* BOLD CMR at 3T in 46 patients with known or suspected CAD [31]. Their BOLD measurements at rest revealed significantly lower T2* values for ischemic segments (27 ± 12ms) compared to normal segments (32 ± 1ms; p < 0.0001) and non-ischemic segments (31 ± 12ms; p = 0.0003). During adenosine stress, T2* values demonstrated a significant increase in normal segments only (37 ± 15ms; p < 0.0001 compared to rest). In contrast, T2* values of non-ischemic (33 ± 15ms; p = 0.19) and ischemic segments (27 ± 12ms; p = 0.06) were not significantly different from rest values. Using a cut-off value of 33.8ms, sensitivity and specificity for the detection of ≥50% angiographic stenosis for rest and stress were 78% and 21%, and 78% and 68%, respectively. Image quality was not consistent and was graded as moderate or poor in about 25% of patients. To overcome these problems with image quality and T2* BOLD methods, several human studies have used T2-prepared steady-state free-precession sequences for oxygenation-sensitive imaging at 1.5 and 3 Tesla [32-34]. Karamitsos and colleagues validated oxygenation-sensitive CMR against myocardial perfusion assessed by positron emission tomography (PET) [32]. They studied 22 patients with single or two-vessel CAD and 10 normal volunteers (Figure 4) and found that BOLD CMR and PET agreed on the presence or absence of ischemia in 18 of the 22 patients (82%) and in all normal subjects. However, per-segment analysis revealed that 40% of myocardial segments with stress myocardial blood flow below the prospectively defined cutoff of 2.45 mL/min/g did not show deoxygenation, whereas the majority of segments with normal perfusion also had normal oxygenation measurements. The investigators concluded that regional myocardial perfusion and oxygenation may be dissociated, indicating that in patients with CAD, reduced perfusion does not always lead to deoxygenation and ischemia. The same group compared oxygenation-sensitive CMR using a T2-prep sequence against 3T quantitative first-pass perfusion during vasodilator stress in 60 patients with suspected CAD referred for diagnostic angiography [35]. Prospective evaluation of BOLD imaging yielded an accuracy of 84%, a sensitivity of 92%, and a specificity of 72% for detecting myocardial ischemia on perfusion CMR and 86%, 92%, and 72%, respectively, for identifying significant coronary stenosis on quantitative coronary angiography. Segment-based analysis revealed evidence of dissociation between oxygenation and perfusion (r = -0.26), with a weaker correlation of quantitative coronary angiography with myocardial oxygenation (r = -0.20) than with perfusion (r = -0.40; p = 0.005 for difference). This study confirmed that myocardial hypoperfusion is not necessarily commensurate with deoxygenation and showed that BOLD-CMR achieves favorable accuracy for identifying the anatomic and functional significance of CAD in a clinical setting. In a similar study, Jahnke and colleagues applied oxygenation-sensitive CMR at baseline and during adenosine infusion and compared results with those acquired by a standard first-pass perfusion CMR protocol during the same session and with quantitative coronary angiography, using 50% stenosis as a cutoff to define relevant stenosis [36]. In contrast to previous studies which used breath-hold sequences, the investigators used a navigator-guided free-breathing T2-prepared segmented gradient echo sequence (Figure 5). They reported a sensitivity and specificity of 85.0% and 80.5%, respectively, compared to quantitative angiography, which is consistent with previous BOLD studies at 3T. Recently oxygenation-sensitive CMR was validated against fractional flow reserve (FFR) in patients with CAD. Walcher et al. found that segments subtended by coronary vessels with an abnormal FFR had reduced BOLD SI change upon hyperemia compared to segments with FFR > 0.8 [37].

The clinical studies which used oxygenation-sensitive CMR to evaluate patients with ischemic and non-ischemic heart disease are summarized in Table 1.

**Table 1:** Clinical studies using oxygenation-sensitive CMR to evaluate ischemic and non-ischemic heart diseases

**Study**	**Field strength**	**BOLD technique**	**Number of participants**	**Investigations**	**Main findings**
***Studies in CAD cohorts***					
Wacker et al. [29]	1.5 T	T2* (ms)	N = 16 patients with single vessel CAD	• BOLD-CMR at rest and during dypiridamole stress	T2* was significantly lower in myocardial segments subtended by stenosed arteries compared to remote myocardium at rest. This difference in T2* increased during dipyridamole stress
			N = 16 healthy volunteers	• Coronary angiography	
Friedrich et al. [30]	1.5 T	T2* (SI)	N = 25 patients with exertional angina	• BOLD-CMR at rest and during adenosine stress	During adenosine, a mean signal intensity decrease was observed for myocardial segments related to coronary stenoses >75%. A non-significant increase was observed in the other segments. Using BOLD signal intensity increase cutoff value of 1.2%, BOLD-CMR had a sensitivity of 88% and a specificity of 47% to correctly classify severe stenoses. BOLD-CMR compared favorably with thallium SPECT
				• Adenosine stress thallium SPECT	
				• Coronary angiography	
Bernhardt et al. [33]	1.5 T	T2 (SI)	N = 46 patients with suspected CAD	• BOLD-CMR at rest and during adenosine stress	BOLD SI change was significantly lower in segments with perfusion deficits compared to patients with visually normal perfusion
				• First-pass perfusion CMR at rest and during adenosine stress	
Manka et al. [31]	3 T	T2* (ms)	N = 46 patients with known or suspected CAD	• BOLD-CMR at rest and during adenosine stress	BOLD CMR at rest revealed significantly lower T2* values for segments subtended by >50% stenosed vessels compared to segments subtended by non-stenosed vessels. Under adenosine T2* values increased only in normal segments
				• Quantitative coronary angiography	
Karamitsos et al. [32]	3 T	T2 (SI)	N = 22 patients with single or two-vessel CAD	• BOLD-CMR at rest and during adenosine stress	BOLD CMR and PET agreed on the presence or absence of ischemia in 18 of the 22 patients
			N = 10 healthy volunteers		(82%) and in all normal subjects. On a per-segment analysis, 40% of myocardial segments with hypoperfusion on PET did not show deoxygenation, whereas the majority of segments with normal perfusion also had normal oxygenation.
				• PET with oxygen-15 labeled water at rest and during adenosine stress	
				• Quantitative coronary angiography	
Arnold et al. [35]	3 T	T2 (SI)	N = 25 CAD patients and N = 20 healthy volunteers (derivation arm)	• BOLD-CMR at rest and during adenosine stress	Prospective evaluation of BOLD imaging yielded an accuracy of 84%, a sensitivity of 92%, and a specificity of 72% for detecting myocardial ischemia and 86%, 92%, and 72%, respectively, for identifying significant coronary stenosis. Segment-based analysis revealed evidence of dissociation between oxygenation and perfusion (r = -0.26).
			N = 60 patients with suspected CAD (prospective arm)	• First-pass perfusion CMR at rest and during adenosine stress (absolute quantification of myocardial blood flow)	
				• Quantitative coronary angiography	
Jahnke et al. [36]	3 T	T2 (SI)	N = 50 patients with suspected or known CAD	• BOLD-CMR at rest and during adenosine stress	The ΔSI measurements differed significantly between normal myocardium, myocardium subtended by a stenosed coronary artery, and infarcted myocardium. A cutoff value of ΔSI = 2.7% resulted in a sensitivity and specificity of 85.0% and 80.5%, respectively to detect coronary artery stenosis. BOLD-ΔSI correlated significantly with the degree of coronary stenosis (r = -0.65, p < 0.001).
				• First-pass perfusion CMR at rest and during adenosine stress (semi-quantitative assessment)	
				• Quantitative coronary angiography	
Walcher et al. [37]	1.5 T	T2 (SI)	N = 36 patients with suspected CAD	• BOLD-CMR at rest and during adenosine stress	Relative BOLD SI increase was significantly lower in myocardial segments supplied by coronary arteries with an FFR ≤ 0.8 compared with segments with an FFR > 0.8
				• Invasive Fractional Flow Reserve (FFR)	
***Studies in non-CAD cohorts***					
Beache et al. [39]	1.5 T	R2* (s)	N = 10 patients with hypertension	• BOLD-CMR at rest and during adenosine stress	Significantly reduced dipyridamole-induced change in the apparent transverse relaxation rate (R2*) in hypertensive patients compared to controls
			N = 9 healthy volunteers		
Karamitsos et al. [34]	3 T	T2 (SI)	N = 18 patients with Syndrome X	• BOLD-CMR at rest and during adenosine stress	No differences in myocardial perfusion and oxygenation between Syndrome X patients and controls
			N = 14 healthy volunteers	• First-pass perfusion CMR at rest and during adenosine stress (absolute quantification of myocardial blood flow)	
				• Quantitative coronary angiography	
Karamitsos et al. [40]	3 T	T2 (SI)	N = 27 patients with overt HCM	• BOLD-CMR at rest and during adenosine stress	MPRI was significantly reduced in HCM compared to controls and athletes, but remained normal in HCM mutation carriers without LVH. Oxygenation response was attenuated in overt HCM compared to controls and athletes. Interestingly, HCM mutation carriers without LVH also showed an impaired oxygenation response to adenosine.
			N = 10 HCM mutation carriers without LVH	• First-pass perfusion CMR at rest and during adenosine stress (semi-quantitative measurement of myocardial perfusion reserve index-MPRI)	
			N = 11 athletes		
			N = 20 healthy volunteers		

*BOLD*, blood-oxygen level dependent; *CAD*, coronary artery disease; *CMR*, cardiovascular magnetic resonance; *FFR*, fractional flow reserve; HCM, hypertrophic cardiomyopathy; *LVH*, left ventricular hypertrophy; *MPRI*, myocardial perfusion reserve index; *PET*, positron emission tomography; *SI*, signal intensity; *SPECT*, single-photon emission computed tomography.

### Clinical potential

**Figure 6:**
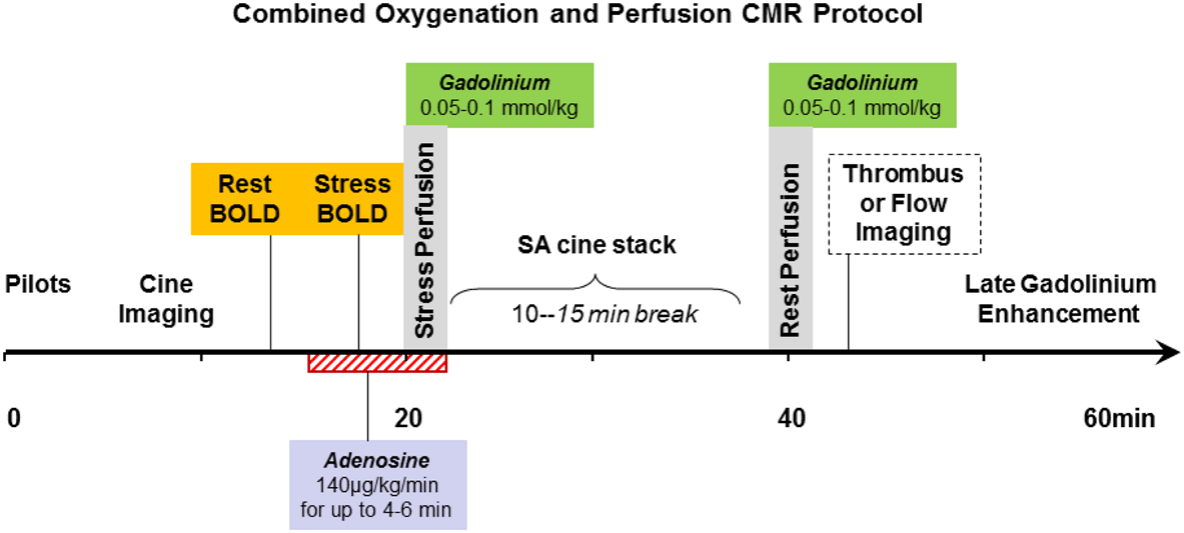
**Single-session CMR examination that allows a comprehensive evaluation of oxygenation and contrast-enhanced first pass perfusion imaging at rest and during adenosine stress.** Standard parts of the protocol are cine imaging and late gadolinium enhancement CMR. Thrombus and flow imaging are optional components of the protocol. The total duration of the protocol is <60min. BOLD, blood-oxygen level-dependent; CMR, cardiovascular magnetic resonance; SA, short-axis.

The relationship between regional myocardial blood flow and epicardial CAD is multifaceted. Oxygen supply and demand can individually vary in response to physiological stimuli, resulting in differing magnitudes of myocardial ischemia that may not be fully discernable via assessment of perfusion alone. Whilst conceptually impairment of both perfusion and oxygenation occur early in the ischemic cascade, they may not always occur simultaneously in the CAD cohort. It is therefore conceivable that, the addition of oxygenation assessment (using oxygenation-sensitive CMR) to the non-invasive imaging armamentarium will improve our ability to detect functionally significant coronary stenoses. An additional advantage of oxygenation-sensitive CMR compared to perfusion CMR techniques is the detection of inducible ischemia without the use of exogenous contrast which is important for patients with severely impaired renal function to whom administration of gadolinium is problematic. Furthermore, oxygenation-sensitive CMR can provide interesting mechanistic insights and, thus, offer an improved pathophysiological understanding of diseases that affect the myocardial microvasculature. Recent human studies support these preclinical findings. In patients with 1 or 2-vessel CAD, oxygenation signal intensity changes during hyperemia in ‘remote to ischemia’ segments are intermediate compared to segments of stenosed and normal coronary arteries. This finding probably reflects the presence of diffuse microvascular dysfunction. The ability of oxygenation-sensitive CMR to detect microcirculatory changes has also been shown in patients with hypertension [39] and in patients with hypertrophic cardiomyopathy including mutation carriers without left ventricular hypertrophy [40]. Another recent study showed that patients with Syndrome X (chest pain, abnormal stress test and normal coronary arteries on angiography) have no evidence of hypoperfusion or deoxygenation using first-pass perfusion CMR and BOLD imaging at 3T, respectively [34]. A particularly compelling aspect of oxygenation-sensitive CMR is the ability to detect deoxygenation in addition to existing CMR techniques which provide surrogates of ischemia (regional function, first-pass perfusion during vasodilator stress). BOLD imaging can be a straightforward addition on current CMR protocols in patients with known or suspected CAD undergoing vasodilator stress. A suggested CMR protocol that combines stress CMR perfusion and oxygenation assessment is shown in Figure 6.

### Current limitations and strategies to overcome them

**Figure 7:**
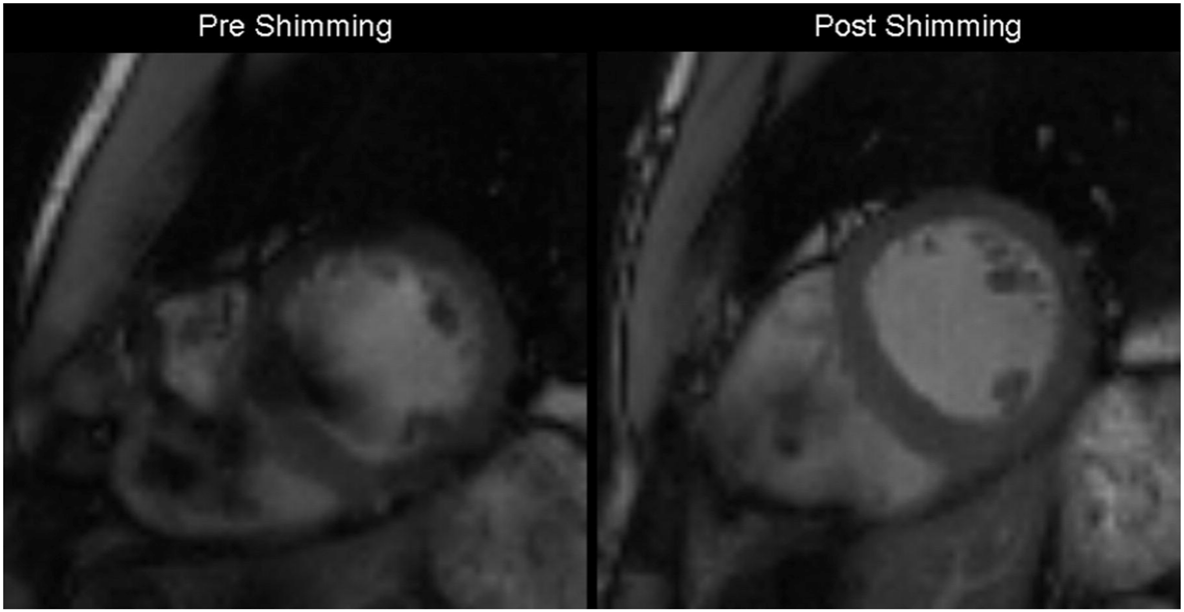
**An example of the effect of shimming (correction of inhomogeneities in the magnetic field) in BOLD images.** The image on the left is acquired before shimming and shows significant artifacts in the septum, the anterior wall, and the blood pool. After shimming adjustments (right image), image quality is significantly improved. From [32].

While the recent advances in oxygenation-sensitive imaging in human and animal studies are promising, oxygenation-sensitive CMR is still in the steep part of a learning curve and a widespread clinical application will require fine-tuned simplified protocols and evaluation tools [41]. Although the feasibility of the technique at 1.5 T has been demonstrated, implementation of the cardiac BOLD approach at 1.5T is fundamentally limited by the relatively small difference in the signal between normal and de-oxygenated myocardial regions. At 3T, the blood and extravascular tissue T2 is much more sensitive to its oxygenation level than at 1.5 T, consequently contrast will be increased [8,42]. This boost in contrast is accompanied by the increased signal to noise ratio at higher field strength, which makes 3 Tesla an attractive field strength for cardiac BOLD imaging. However, the increased signal comes at the expense of artifacts from inhomogeneities of the magnetic field and careful shimming is essential at 3 Tesla, particularly for SSFP sequences (Figure 7). Furthermore, artifacts may occur in the inferolateral wall due to the great cardiac vein or the heart-lung interface. Oxygenation-sensitive imaging has not been tested adequately in patients with coronary stents which are also likely to cause artifacts. There is also uncertainty as to which is the best approach for BOLD-CMR, i.e. measurement of T2* or signal intensity changes? Although T2* based methods appear to be more sensitive, they are also more susceptible to artifacts and implementation of T2* imaging at 3T may be particularly challenging.

### Future directions

**Figure 8:**
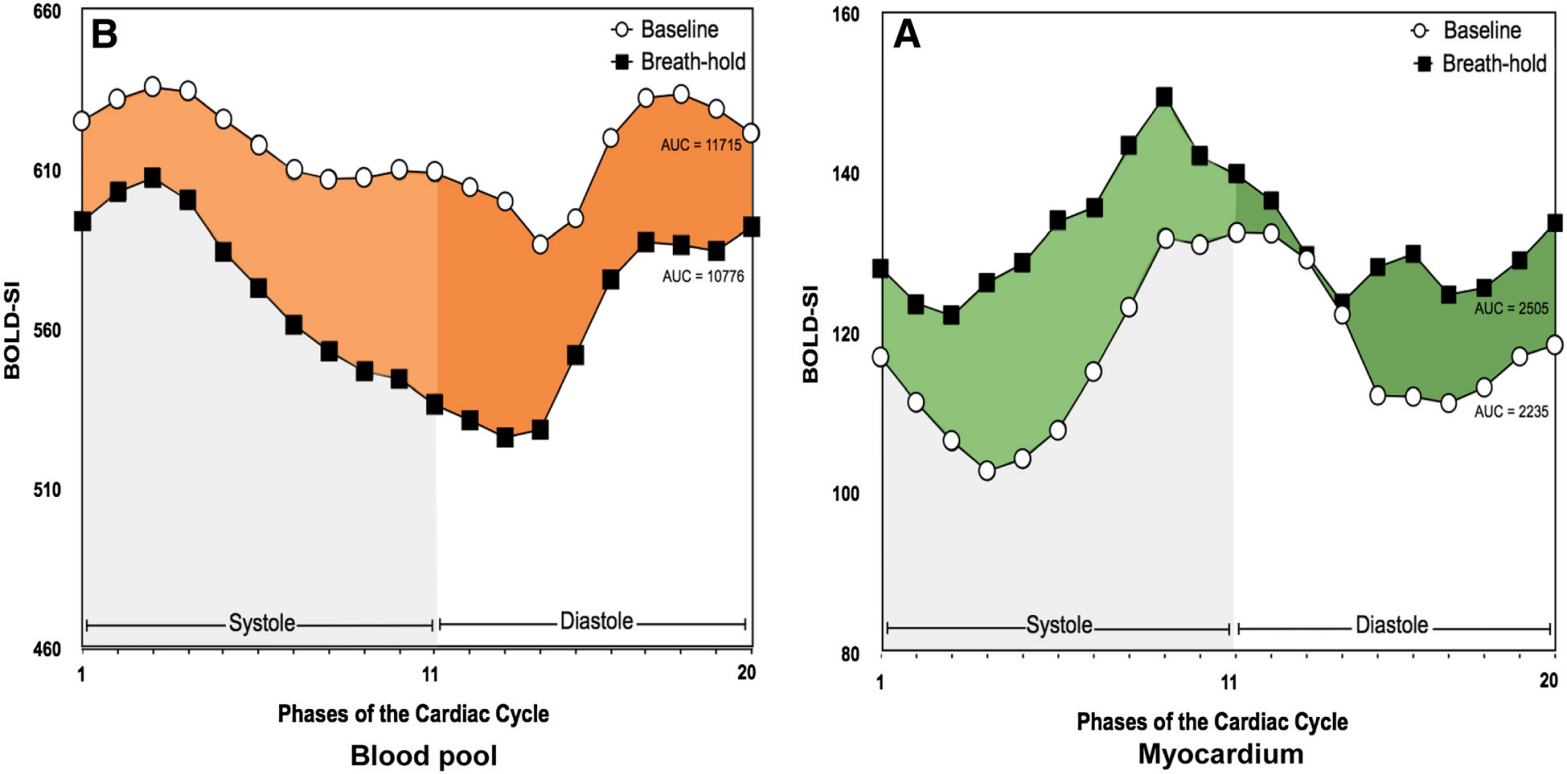
**Oxygenation changes throughout the cardiac cycle as assessed by CMR during apnea in the blood (left) and the myocardium (right).** While the signal intensity in the blood drops in the blood (de-oxygenation), it increases in the myocardium due to increased myocardial blood flow. Measurements were performed in the same images (modified from [23].

Current challenges of oxygenation-sensitive imaging are mainly due to its limited contrast-to-noise and thus, future developments will have to focus on improving the robustness of the CMR protocols and work on strategies to maximize detectable changes by improving stimuli and data acquisition. Such strategies include higher field strength [8], modifications of CMR sequences [43], improved methods for defining the area of interest [38]and novel ways for diagnostic changes of oxygenation [23] (Figure 8). Furthermore, signal-intensity based methods will have to be compared with T2* mapping approaches.

In addition to refining techniques, pre-clinical and clinical research is required to reproduce published data.

## Conclusion

Despite current shortcomings, oxygenation-sensitive CMR has passed the stage of an experimental attempt to become a tool which awaits serious assessment of its clinical utility in clinical scenarios. Tissue oxygenation as a true biomarker, as assessed by a very safe, non-invasive, contrast-free technique, in combination with other cardiac markers may become a major diagnostic target in patients with suspected coronary artery disease but also for primary or secondary microvascular disease.

## Competing interests

Dr Karamitsos has no competing interests to report. Matthias G. Friedrich holds a pending patent on “Inducing and Measuring Myocardial Oxygenation Changes as a Marker for Heart Disease” (US Pat. Serial No. 61/680,981).

## Authors’ contributions

MGF and TDK drafted the manuscript and figures, read and approved the final manuscript.
